# Detached Anterior Horn of the Medial Meniscus Mimicking a Parameniscal Cyst

**DOI:** 10.1155/2015/706241

**Published:** 2015-10-15

**Authors:** Shoji Fukuta, Takahiko Tsutsui, Tetsuya Matsuura, Naoto Suzue, Daisuke Hamada, Tomohiro Goto, Koichi Sairyo

**Affiliations:** Department of Orthopedics, Tokushima University, 3-18-15 Kuramoto-cho, Tokushima 7708503, Japan

## Abstract

We report a case of a detached anterior horn of the medial meniscus with anterior knee pain. Preoperative magnetic resonance images of the knee were initially interpreted as a parameniscal cyst. Arthroscopic examination revealed subluxation of the anterior horn of the medial meniscus due to detachment from its anterior tibial insertion. Arthroscopic fixation with a suture anchor was successful and the cystic lesion was no longer visible on postoperative images.

## 1. Introduction

Detachment of the anterior horn of the medial meniscus from the tibial insertion is considered to be a rare pathology of the knee. Several case series of this type of meniscal injury have been previously reported; however, the diagnosis and treatment of this pathology have not been well established. We encountered a case involving a symptomatic detached anterior horn of the medial meniscus, diagnosed initially as a parameniscal cyst based on preoperative magnetic resonance images. Arthroscopic repair using a suture anchor was successful. In this case report, we recommend that clinicians should be aware of this pathology and take it into consideration as a possible cause of anterior knee pain.

## 2. Case Report

A 34-year-old woman presented with a 3-month history of pain in the left knee with no history of trauma. The pain was aggravated on ascending or descending the stairs. On physical examination, her left knee had marked tenderness along the anteromedial joint line. The range of movement was not limited. McMurray's test was negative and the knee was stable with a negative Lachman test and a negative pivot shift test.

**Figure 1:**
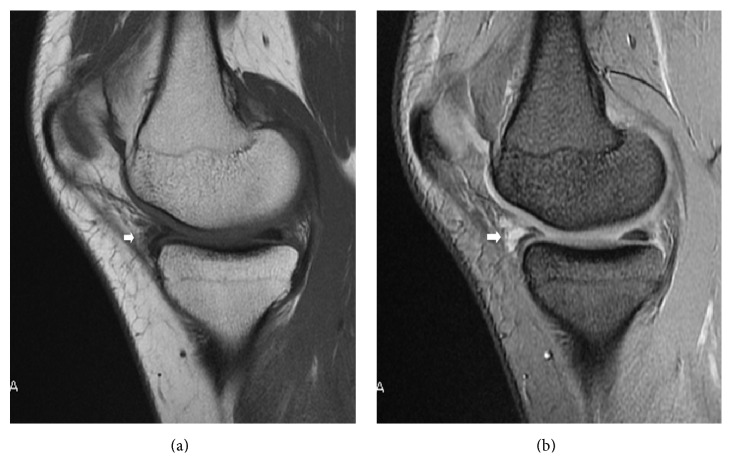
Preoperative magnetic resonance images of the right knee showed a cystic lesion adjacent to the anterior horn of the medial meniscus. (a) Sagittal T1-weighted image and (b) Sagittal T2-weighted image. White arrows indicate the lesion.

On plain radiographs no degenerative changes were visible in the medial compartment. Magnetic resonance images of the right knee showed a cystic lesion adjacent to the anterior horn of the medial meniscus (Figure 1). No signal abnormalities were found in the body of the medial meniscus or in other structures. These findings on preoperative MRI of the knee were initially interpreted as a parameniscal cyst.

**Figure 2:**
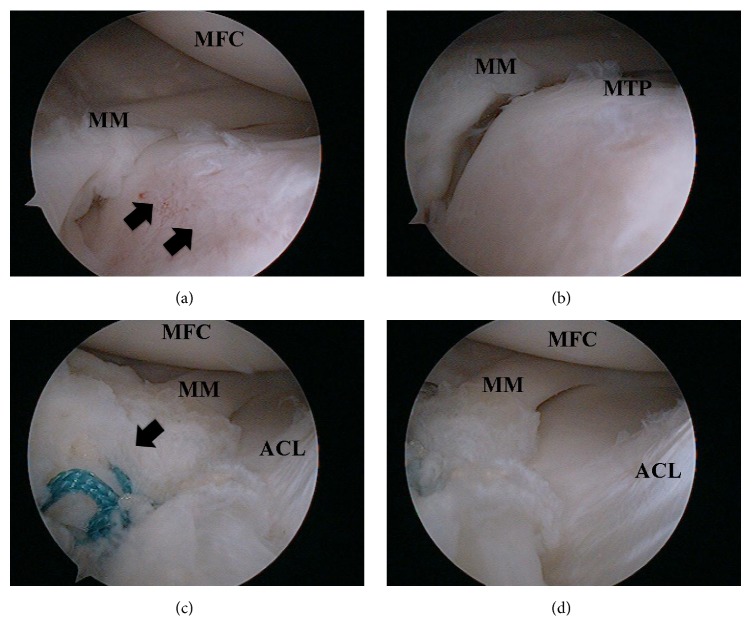
Arthroscopic views. (a) The anterior horn of the medial meniscus was detached and the anterior tibial surface was exposed (black arrow). (b) The anterior portion of the medial meniscus was subluxated with the knee flexed at 45 degrees. (c) The detached anterior horn was fixed to the tibial surface with a suture anchor (black arrow). (d) The anterior portion of the medial meniscus was not subluxated with the knee flexed at 45 degrees. MFC: medial femoral condyle, MM: medial meniscus, MTP: medial tibial plateau, and ACL: anterior cruciate ligament.

The patient underwent arthroscopy of her right knee under general anesthesia through routine medial and lateral infrapatellar portals. On arthroscopic examination, the knee was found to have a detached anterior horn of the medial meniscus (Figure 2(a)). The anterior one-third of the meniscus was hypermobile and was displaced below the medial tibial plateau with the knee flexed about 45 degrees (Figure 2(b)). No parameniscal cyst was detected around the anterior horn of the medial meniscus and the ACL was intact. After refreshing the original footprint of the anterior horn on the anterior tibia using a motorized shaver, a bioabsorbable suture anchor single-loaded with nonabsorbable suture (Panalok Loop, Depuy-Mitek, Norwood, MA) was inserted through the regular medial portal. Each limb of the suture was passed through the meniscus by a suture passer. Horizontal mattress suture fixation was secured by arthroscopic knot-tying techniques (Figure 2(c)). After this procedure, stability of the meniscal body was restored (Figure 2(d)).

**Figure 3:**
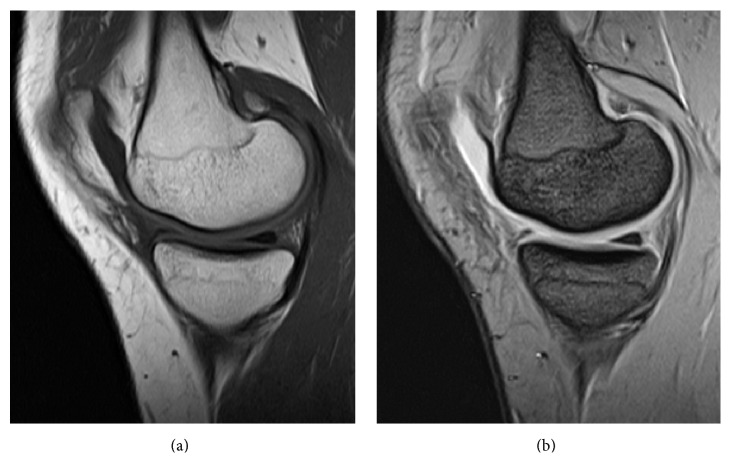
No abnormal signals adjacent to the anterior horn of the medial meniscus were noted on magnetic resonance images at 12 months postoperatively.

Postoperatively, the right knee was immobilized in full extension for 3 weeks, followed by active and passive range of movement exercise. The patient was followed up regularly. At the final follow-up at 12 months postoperatively, her right knee was free of symptoms and MRI showed no cystic lesions adjacent to the anterior horn (Figure 3).

## 3. Discussion

Symptomatic unstable anterior horn of the medial meniscus was first mentioned by Jones [1] in 1925. Boucher [2] reported that 34 knees had strain or rupture of the anterior marginal attachment in 350 knee arthrotomies for suspected internal derangement; however, he did not comment on treatment for this injury. There have been few case studies that described arthroscopic findings and clinical outcomes. Clancy et al. [3] described the cases of 13 patients with symptomatic dislocation of the anterior horn of the medial meniscus. They performed total meniscectomies in the first four patients, meniscal repair in the next four patients, and arthroscopic partial meniscectomies in the last five patients. They reported that 11 of the 13 patients had excellent final results.

In contrast Pinar et al. [4] obtained satisfactory results of 15 dislocations of the anterior horn of the medial meniscus without any surgical treatments. They concluded that a dislocating anterior horn of the medial meniscus was a normal anatomic variant with little or no clinical significance. In their series, however, only 2 of 15 knees had isolated lesions and the other 13 had associated lesions.

The anterior horn of the medial meniscus is normally attached to the anterior surface of the tibia; however, several anatomic variations of this attachment have been reported [5, 6]. The absent anterior insertion to the tibia induces abnormal mobility in the anterior horn and may cause anterior knee pain. Damage to secondary supporting structures such as the transverse ligament may also increase instability of the medial meniscus.

The formation of the meniscoligamentous complex is well established in the eight-week embryologic development [7] and the anterior horn of the medial meniscus attaches to the anterior aspect of the tibia during the 10th and 11th weeks of embryo [8]. Collagen fibers are later organized in a transverse and circumferential fashion. It is not clear at which stage of embryologic development anatomical variations of the insertion occur. Ohkoshi et al. [5] classified variants of the attachment into four categories based on arthroscopic findings of 953 knees. They reported that 10.9% of the patients had variants of the attachment with hypermobility of the medial meniscus. Several cadaveric studies also showed similar anatomic variation of the attachment of the anterior horn of the medial meniscus [6].

Clinically, it is not easy to differentiate a traumatic instability from an anatomic variant. Lesions of the medial meniscus are most frequently seen on the posterior portion of the meniscal body so that little attention may be paid to the anterior horn. Furthermore, the anterior horn of the medial meniscus is often covered with a synovial tissue and may be missed during routine arthroscopic examination. In this case, complete arthroscopic examination was performed around the anterior horn due to preoperative misdiagnosis as a parameniscal cyst on MRI. To avoid unnecessary fixation of the normal variants, exclusion of any other lesions is important. Surgical treatment can be an option for isolated detachment of the anterior horn when conservative treatment has failed.

Arthroscopic repair of the anterior horn of the medial meniscus is more feasible than partial meniscectomy. Recent development of arthroscopic devices makes arthroscopic fixation with a suture anchor easier. In this case we used the same technique that is used to repair tears of the superior labrum anterior and posterior lesions of the shoulder. This technique is less invasive than a pull-out technique. Using a knotless suture, anchor is also an option for arthroscopic fixation [9].
